# An Autocrine Wnt5a Loop Promotes NF-κB Pathway Activation and Cytokine/Chemokine Secretion in Melanoma

**DOI:** 10.3390/cells8091060

**Published:** 2019-09-10

**Authors:** Gastón Barbero, María Victoria Castro, María Belén Villanueva, María Josefina Quezada, Natalia Brenda Fernández, Sharon DeMorrow, Pablo Lopez-Bergami

**Affiliations:** 1 Centro de Estudios Biomédicos, Biotecnológicos, Ambientales y Diagnóstico (CEBBAD), Universidad Maimónides, Buenos Aires C1405, Argentina; 2 Consejo Nacional de Investigaciones Científicas y Técnicas (CONICET), Buenos Aires C1425, Argentina; 3 Instituto de Biología y Medicina Experimental, Buenos Aires C1428, Argentina; 4 Division of Pharmacology and Toxicology, College of Pharmacy, The University of Texas at Austin, Austin, TX 78712, USA; 5 Department of Internal Medicine, Dell Medical School. The University of Texas at Austin, Austin, TX 78712, USA; 6 Central Texas Veterans Healthcare System, Austin, TX 78712, USA

**Keywords:** Wnt5a, NF-κB, Akt, melanoma, cytokine, chemokine

## Abstract

Wnt5a signaling has been implicated in the progression of cancer by regulating multiple cellular processes, largely migration and invasion, epithelial-mesenchymal transition (EMT), and metastasis. Since Wnt5a signaling has also been involved in inflammatory processes in infectious and inflammatory diseases, we addressed the role of Wnt5a in regulating NF-κB, a pivotal mediator of inflammatory responses, in the context of cancer. The treatment of melanoma cells with Wnt5a induced phosphorylation of the NF-κB subunit p65 as well as IKK phosphorylation and IκB degradation. By using cDNA overexpression, RNA interference, and dominant negative mutants we determined that ROR1, Dvl2, and Akt (from the Wnt5a pathway) and TRAF2 and RIP (from the NF-κB pathway) are required for the Wnt5a/NF-κB crosstalk. Wnt5a also induced p65 nuclear translocation and increased NF-κB activity as evidenced by reporter assays and a NF-κB-specific upregulation of RelB, Bcl-2, and Cyclin D1. Further, stimulation of melanoma cells with Wnt5a increased the secretion of cytokines and chemokines, including IL-6, IL-8, IL-11, and IL-6 soluble receptor, MCP-1, and TNF soluble receptor I. The inhibition of endogenous Wnt5a demonstrated that an autocrine Wnt5a loop is a major regulator of the NF-κB pathway in melanoma. Taken together, these results indicate that Wnt5a activates the NF-κB pathway and has an immunomodulatory effect on melanoma through the secretion of cytokines and chemokines.

## 1. Introduction

Melanoma is the fifth and sixth most common cancer for men and women, respectively, and thus represents a major public health problem [1,2]. In addition to its high incidence, melanoma is one of the most aggressive tumor types with a 5-year survival rate of around 20% [1,2]. Immunotherapy and molecular-targeted therapies have shown promise in the management of this cancer, and currently there are several single or combination therapies approved for first-line treatment of metastatic or unresectable diseases. Immune checkpoint inhibitors (ICI), such as anti-PD-1 and anti-CTL4, provide durable responses, but around half of the patients present innate resistance to these treatments. Targeted therapies against BRAF and MEK have achieved benefits in both progression-free and overall survival and a pronounced reduction in tumor burden [3]. However, the duration of response is limited by acquired resistance to therapy [4]. For these reasons, there is an unmet need to identify new molecular targets to use either in combinatorial approaches or as second-line therapy.

In this work, we focused on Wnt5a, an extracellular ligand that has been implicated in cancer progression [5]. Wnt5a is a member of the Wnt large family of lipid-modified glycoproteins and transduces its signal upon binding to different plasma membrane receptors or co-receptor complexes and upon activation of Dishevelled (Dvl) phosphoproteins in the cytoplasm [6]. Wnt5a activates several signaling pathways, collectively named “non-canonical”, since they are independent of β-catenin, the critical intermediate of the Wnt canonical pathway. The Wnt5a cascades are diverse, and downstream pathways implicating cGMP-Ca^++^, Rap1, JNK, PKA, GSK3, PKC, Src, mTOR, and Akt have been described [6,7]. Besides its crucial role in embryonic development, deregulation of Wnt5a has been implicated in several human diseases, most importantly cancer [5,8,9]. However, the biological functions of non-canonical Wnt signaling in tumorigenesis are far from being elucidated. Wnt5a is undoubtedly the most studied non-canonical Wnt ligand in cancer. Previous studies have demonstrated that Wnt5a has a dual effect on the progression of various types of human cancer [10], indicating that this pathway is particularly dependent on the cellular context [7]. Wnt5a is considered to have a tumor-suppressive function in neuroblastoma [11], leukemia [12], ductal breast carcinomas [13], ER-positive breast cancer [14], colorectal cancer [15], and thyroid cancer [16]. On the other hand, a protumorigenic role for Wnt5a has been shown for T-cell leukemia [17], melanoma [18], gastric cancer [19], non-small-cell lung cancer [20], pancreatic cancer [21], and prostate cancer [22]. The tumor-promoting activities of Wnt5a affect multiple cellular processes, including proliferation, differentiation, angiogenesis, chemoresistance, migration, invasion, epithelial-mesenchymal transition (EMT), and metastasis [5]. In melanoma, Wnt5a positively regulates many of these processes and has been signaled as a promising therapeutic target [23].

It has been proposed that, besides the eight “hallmarks of cancer”, cancer cells have two traits, namely genome instability and inflammation, which are key to fostering many of those “hallmarks” [24]. Intriguingly, although Wnt5a regulates inflammatory processes in both infectious and inflammatory diseases, such as tuberculosis, sepsis, psoriasis, rheumatoid arthritis, and atherosclerosis [25,26], its role in inflammation in the context of cancer has been poorly investigated. Wnt5a was shown to induce the expression of cytokines at the mRNA [27] and protein levels [28] in MKN-7 gastric carcinoma cells and SKOV-3 ovarian carcinoma cells, respectively. However, these studies did not explore the mechanisms underlying cytokine regulation. Since inflammation is a critical factor for cancer progression, it is of utmost importance to better understand the role of Wnt5a in this process. We reasoned that a meaningful approach to achieve this goal is to study the effect of Wnt5a on the activation of NF-κB, a pivotal mediator of inflammatory responses [29]. The NF-κB pathway is upregulated in advanced melanoma as a consequence of various mechanisms, where the relative relevance of each of them has not been determined [30]. NF-κB is a heterodimeric transcription factor formed by the proteins RelA (or p65), RelB, c-Rel, NF-κB1 (or p50), and NF-κB2 (or p52), which share an N-terminal Rel Homology Domain, which is key to the dimerization and union to DNA. NF-κB subunits are sequestered in the cytoplasmic compartment of normal cells through its association with members of the IκB family (IκBα, IκBβ, and IκBε) [30,31]. Stimulatory signals activating the membrane receptors BCR and TNFR and several extracellular stimuli (inflammatory cytokines, viral and bacterial infections, oxidative and DNA-damaging agents, ultraviolet light, and osmotic shocks) activate NF-κB through different mechanisms, classified as canonical, non-canonical, and alternative. The canonical pathway is activated by TNFα or IL-1β and relies on the activation of IKK, the degradation of IκBα, and the translocation of the p65/p50 dimer to the nucleus where it regulates gene expression. The non-canonical pathway is activated by CD40 or RANKL and induces an IKKα-dependent phosphorylation and cleavage of the p100 form of NF-κB2 into p52, which binds to RelB and translocates to the nucleus. NF-κB targets inflammation by inducing the expression of various pro-inflammatory genes, including those encoding cytokines, chemokines, and adhesion molecules [29,30,31,32]. Of note, activation of NF-κB by Wnt5a has been shown in different cellular contexts but has been poorly studied in cancer cells. In the present study, we provide a comprehensive description of the signaling proteins that are activated by Wnt5a and result in NF-κB activation and upregulation of cytokine secretion in melanoma.

## 2. Materials and Methods

### 2.1. Reagents

The final concentrations of the reagents were: rWnt5a (R&D Systems, Minneapolis, MN, 645-WN/CF), 400 ng/mL; IWP-2 (Cayman Chemicals, Ann Arbor, MI, 04614560-30), 20 µM; hTNFα (R&D Systems, 210-TA-020), 10 µg/mL; Cycloheximide (Sigma, St. Louis, MO, C7698-1G), 10 µg/mL; BAY11-7082 (Cayman Chemicals, 10010266), 10 µM; LiCl (Sigma, L9650), 10 mM; LY294002 (Calbiochem, Burlington, MA, 440202), 10 µM; JSH-23 (Abcam, UK, 144824), 10 µM; Tunicamycin (Cayman Chemicals, 11089-65-9), 4 µM; and Box5 (EMD Millipore, Burlington, MA, 681673), 200 µM.

### 2.2. Cell Culture 

Melanoma cell lines were kindly provided by Dr. Meenhard Herlyn (The Wistar Institute, Philadelphia, PA) and Dr. Zeev Ronai (Sanford Burnham Prebys Medical Discovery Institute, La Jolla, CA). The melanocytic cell line Mel-STV was kindly provided by Dr. Robert Weinberg (Whitehead Institute for Biomedical Research, Cambridge, MA). All cell lines were maintained in DMEM, supplemented with 10% fetal bovine serum (FBS, Gibco, Carlsbad, CA) 100 U/mL penicillin, and 100 mg/mL streptomycin (Invitrogen, Carlsbad, CA) at 37 °C and 5% CO_2_. Cells were transfected with calcium phosphate or by a Lipofectamine PLUS reagent (Invitrogen), following the manufacturer’s protocol. The cell lines were free of mycoplasma contamination and were authenticated by short tandem repeat (STR) analysis as described [33]. The L-Wnt5a and L control cells were a gift from by Dr. Stuart Aaronson (Mount Sinai School of Medicine, New York, NY, USA). The conditioned media (CM) from these cells were obtained as described by ATCC. Briefly, L cells were seeded at a density of 1 × 10^6^ cells in a 100 mm dish containing DMEM with 1% FCS and cultured for 4 days. CM was then harvested, centrifuged at 1000 *g* for 10 min, filtered, and stored at −20 °C. To stimulate melanoma cells with Wnt5a, cells (1−2 × 10^6^) were seeded in a 100 mm dish and placed into the incubator for 24–48 h. The culture medium was removed and either the Wnt5a conditioned medium (Wnt5a-CM) or control conditioned medium (Control-CM) was added for 30 min, except when indicated. When evaluating the role of a protein kinase, the corresponding inhibitor was added both before and during the stimulation with CM. In these experiments, the cells were pre-incubated with the corresponding compound for 30 min. The culture media was then removed and the CM (containing the inhibitor) was added to the cell for 30 min. In these experiments, DMSO was used as a control.

### 2.3. Constructs

The shRNA against ROR1 was validated in a previous publication from our laboratory [34]. The shRNAs against ROR2 and Wnt5a were described previously [35]. The shRNA against the Rictor corresponds to Addgene plasmid #1854 and has been validated and used in several publications before. The shRNA plasmids targeting Dvl2 and Dvl1-3 (isoforms 1 and 3 were targeted by the same sequence) were kindly ceded by Dr. Stuart Aaronson (Mount Sinai School of Medicine, New York) and have been previously validated [36,37]. The plasmids encoding ROR1 and ROR2 were kindly provided by Dr. Luca Grumolato (Universite de Rouen, Rouen, France). The above constructs were cotransfected into HEK-293T cells together with packaging plasmids to generate viral particles. Viral supernatants were harvested, filtered, and used to transduce melanoma cells. In all cases, cells were selected with 3 μg/mL puromycin for one week and then maintained with 1 μg/mL puromycin. The plasmids encoding TRAF-DN and RIP-DN were provided by Dr. Hasemu Habelhah (University of Iowa, Iowa City, IA). Cells transfected with TRAF-DN and RIP-DN were selected with 0.5 mg/mL neomycin for three weeks. 

### 2.4. Western Blotting

For the Western blotting analysis, cell lysates were collected by addition of lysis buffer, supplemented with protease and phosphatase inhibitors for 10 min on ice [38]. The cell lysates were centrifuged at 13,000 rpm for 15 min at 4 °C, and the supernatants were collected and quantified using the Bradford method. Between 20–50 μg of proteins were diluted in a 6× Laemmli buffer, boiled at 95 °C for 5 min, separated on 8–15% SDS-PAGE gels, and then transferred to a nitrocellulose membrane. The membranes were blocked with 5% milk in 0.05% Tween-PBS at room temperature for 1 h and then incubated with the primary antibodies at 4 °C overnight. The following antibodies were used: Akt1 (sc-5298), p-Akt (sc-7985), Bcl2 (sc-7382), Cyclin D (sc-8396), Dvl2 (sc-13974), Dvl3 (sc-8027), GAPDH (sc-25778), IκBα (sc-1643), p65 (sc-37), RelB (sc-226), Rictor (sc-271081), ROR1 (sc-130386), ROR2 (sc-98486), TRAF2 (sc-876), and Vinculin (sc-25336) from Santa Cruz Biotechnologies. Antibodies to Histone 3 (cs-14269), IKKα (cs-2682), p-IKKα/β (cs-2697), p-p65 (cs-3303), and Wnt5a (cs-25305) were from Cell Signaling. Antibodies to Actin (A5441) and Tubulin (T9026) were from Sigma. The corresponding HRP-conjugated secondary antibodies, anti-mouse (GE NA931V) and anti-rabbit (GE NA934), were incubated for 1 h at room temperature. Immunoreactive bands were detected by an ECL system (Amersham Biosciences, UK) using an image reader (ImageQuant 350, GE Healthcare, Chicago, IL). Quantification of band intensities was performed using ImageJ (NIH, Bethesda, MA). The intensity of each band was normalized to GAPDH or other housekeeping genes (i.e., Tubulin or Actin), and the fold change (FC) relative to the control cells was calculated. The band intensities in the phosphoprotein blots were normalized with those of the total proteins obtained from the same blots after stripping and reprobing. To draw a conclusion on a particular experiment, at least three biological (independent) replicates of paired samples were examined to calculate the mean and standard deviation. The log transformation of FC values were calculated to obtain a more symmetric distribution that better suited the normality assumptions of the subsequent t-test. 

### 2.5. Luciferase Assays

M2 cells (3 × 10^5^) were plated into 12-well plates and transfected 24 h later with 0.5 µg of pNF-κB-Luc and pSV40-gal (0.2 µg), using Lipofectamine. At 36 h after transfection, cells were stimulated with Wnt5a-CM and control-CM, and protein samples were prepared at the indicated time points. Luciferase activity was measured using the luciferase assay system (Promega, Madison, WI) in a Berthold luminometer and normalized to the β-galactosidase activity [39] measured in the same sample.

### 2.6. Cell Fractioning

Cell lysates were collected with a Hypertonic Solution (NaCl 500 mM; NaH2PO4 12 mM; NaF 10 mM, Cl2Mg6 5 mM; EDTA 1.5 mM; Igepal NP40, Leupeptin 10 μg/mL; Pepstatin A 5 μg/mL; and Aprotinin 5 μg/mL) and centrifuged for 10 min. Supernatants were collected as the cytoplasmic fraction and pellet was resuspended with a Hypotonic Solution (Hypertonic Solution without NaCl), sonicated five times and centrifuged for 10 min to collect nuclear fraction. 

### 2.7. Cytokine Array

Undiluted supernatants of cells treated with Wnt5a-CM and control CM for 32 h were incubated over night at 4 °C with human inflammation antibody array membranes (Abcam, 134003), a semi-quantitative assay for a simultaneous detection of 40 human inflammatory factors. Capture antibodies were supplied arrayed/spotted on a membrane, with each pair of spots representing a different analyte. After washing with PBS, the membranes were incubated with paired biotinylated detector antibodies and streptavidin HRP, following the manufacturer protocol. Immunoreactive spots were detected by an ECL system (Amersham Biosciences) using an image reader (ImageQuant 350, GE Healthcare). Quantification of band intensities was performed using ImageJ (NIH). The data were analyzed as described in the figure legend. 

### 2.8. ELISA

A Human IL-8 ELISA Set from BD Biosciences (555244) was performed on culture supernatants from cells stimulated with Wnt5a for different times, according to manufacturer protocol. The culture supernatants were assayed at a dilution within the linear range of the IL-8 standards, and the concentration of IL-8 in each sample was determined using a standard curve, as indicated by the kit. 

### 2.9. Immunofluorescence

Cells were seeded in slides and fixed in DMEM with 3% PFA for 10′ at RT after treatment with Wnt5a. Slides were washed in PBS and placed in permeabilization solution (containing Triton-X and Sucrose) on ice for 20′. Slides were then blocked in TBS-tween with 3% BSA during 1 h at RT. Next, they were incubated with a p65 antibody for 1 h, and after 3 washes with PBS, they were incubated with a secondary antibody (Alexa Fluor 488) for 1 h in the dark. Finally, slides were mounted with Vectashield DAPI ANTIFADE Mounting Medium. Pictures were taken on Nikon C1 Plus Microscopy and analyzed on FIJI. We calculated a (corrected total cell section fluorescence (CTCF) coefficient adapted from McCloy et al. [40].

### 2.10. Bioinformatics Analysis

The analysis of the expression of Wnt5a and cytokines in human samples was performed using data from The Cancer Genome Atlas (TCGA) project. Expression data from TCGA dataset for skin cutaneous melanoma (SKCM), containing 469 patients, were extracted using cBioportal. The plot figures were generated in Excel. 

### 2.11. Statistics

Except when indicated, experiments were performed at least 3 times. The sample size was determined using the Power and Sample Size Program. All data are expressed as the mean ± SD. Mean differences between groups were determined using student’s t-tests or one-way ANOVAs, followed by post hoc tests. Values of *p* < 0.05 were considered statistically significant. Statistical analyses were conducted using software from Graph-Pad Prism. The number of independent experiments and specific statistical analyses used in each experiment is indicated in the figure legends.

## 3. Results

### 3.1. Wnt5a Induces p65 Phosphorylation in Melanoma

**Figure 1:**
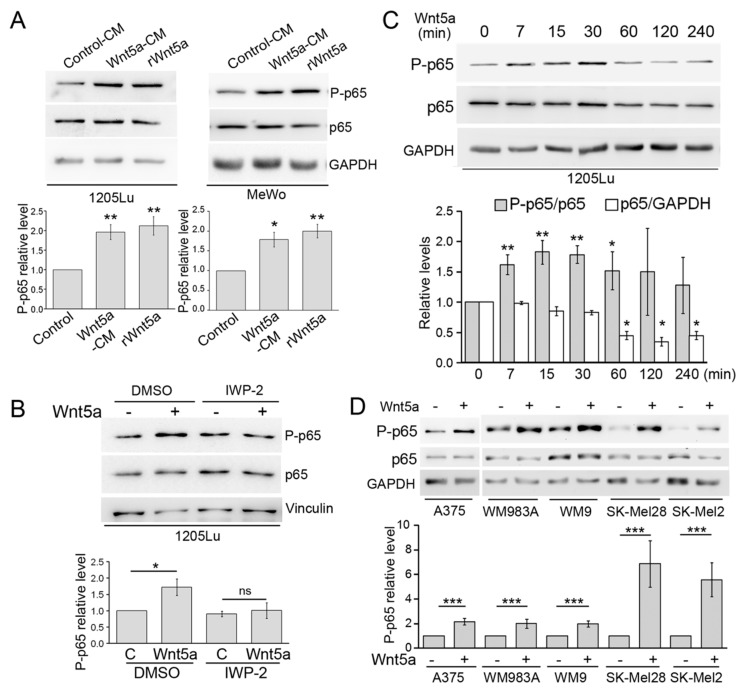
Wnt5a increased p65 phosphorylation. (**A**) 1205Lu and MeWo cells were stimulated for 30 min with recombinant Wnt5a (rWnt5a) or Wnt5a conditioned media (Wnt5a-CM). Protein extracts were blotted with the indicated antibodies. (**B**) 1205Lu cells were stimulated with Wnt5a-CM (Wnt5a +) or Control CM (Wnt5a −), which were collected in the presence of either IWP-2 (20 µM) or DMSO. Protein extracts were blotted with the indicated antibodies. (**C**) 1205Lu cells were stimulated with Wnt5a-CM (Wnt5a) for the indicated times, and the proteins extracts were then blotted with the indicated antibodies. (**D**) The indicated melanoma cell lines were stimulated with Wnt5a-CM (Wnt5a) for 30 min, and the proteins extracts were then blotted with the indicated antibodies. GAPDH or Vinculin were used as loading controls. The blots displayed are representative of three independent experiments. Bar graphs show the mean ± SD (from three independent experiments) of P-p65 levels, normalized to the level of total p65, detected after stripping the membrane. In panel **C**, to evaluate changes in total p65, the data were normalized to GAPDH levels. Results are expressed as the fold change relative to control-treated cells (lane 1 in **A**–**C**). In panel **D**, each cell line has its own control-treated lane. 1205Lu, SK-Mel28, and WM9 are both BRAF and PTEN mutant; A375 and WM983A are BRAF mutant; SK-Mel2 is Ras mutant; MeWo is p53 mutant. The statistical significance was tested by a student’s t-Test (treated sample vs. paired control in B,D) or ANOVA, followed by Dunnett’s Multiple Comparison Test (**A**,**C**), using log transformed fold change (FC) values. * *p* < 0.05, ** *p* < 0.01, *** *p* < 0.001, n = 3.

To assess whether Wnt5a activates the NF-κB pathway, MeWo and 1205Lu human melanoma cells were stimulated with Wnt5a, and the degree of p65 S536 phosphorylation, a marker of NF-κB activation [41], was determined by western blot. Our data show that both recombinant Wnt5a (rWnt5a) and the Wnt5a conditioned medium (Wnt5a-CM) obtained from L-Wnt5a fibroblasts significantly increased p65 phosphorylation compared with the conditioned medium from control L cells (Control-CM) (Figure 1A). To assess the specificity of the effect induced by Wnt5a-CM, the L-Wnt5a cells were incubated with IWP-2. This compound inhibits Porcupine-dependent Wnt5a palmitoylation, a critical step in Wnt5a biosynthesis [42], hence preventing Wnt5a secretion (Figure S1). The conditioned medium obtained from L-Wnt5a cells under this condition failed to phosphorylate p65, confirming the participation of Wnt5a in this process (Figure 1B). Since both rWnt5a and Wnt5a-CM similarly stimulated p65 phosphorylation, we used Wnt5a-CM throughout this work. A time-course experiment revealed that p65 phosphorylation is induced by 7 min and reaches a maximal level between 15 and 30 min after Wnt5a treatment. The increase in P-p65 level was no longer seen 2 h after Wnt5a treatment (Figure 1C). After 1 h of Wnt5a treatment, we observed a slow but steady decrease in total p65 levels, a proposed mechanism of shutting down NF-κB signals (Figure 1C) [43]. To determine whether this circuit linking Wnt5a with NF-κB is prevalent in melanoma, we performed a similar Wnt5a treatment in four additional melanoma cell lines derived from metastasis (A375, WM9, SK-Mel28, and SK-Mel2), a cell line derived from a primary tumor (WM983A), and a melanocyte cell line. These cells also present different genetic backgrounds (see legend). In all cases, Wnt5a elicited a significant rise in p65 phosphorylation with a fold increase ranging from 2 in A375 to 7.2 in SKMel28 (Figure 1D and Figure S2). These results demonstrate that Wnt5a readily phosphorylates p65 throughout the melanocytic lineage and irrespectively of the genetic background.

### 3.2. ROR1, Dvl2, and Akt are Required for Wnt5a-Dependent Activation of NF-κB

**Figure 2:**
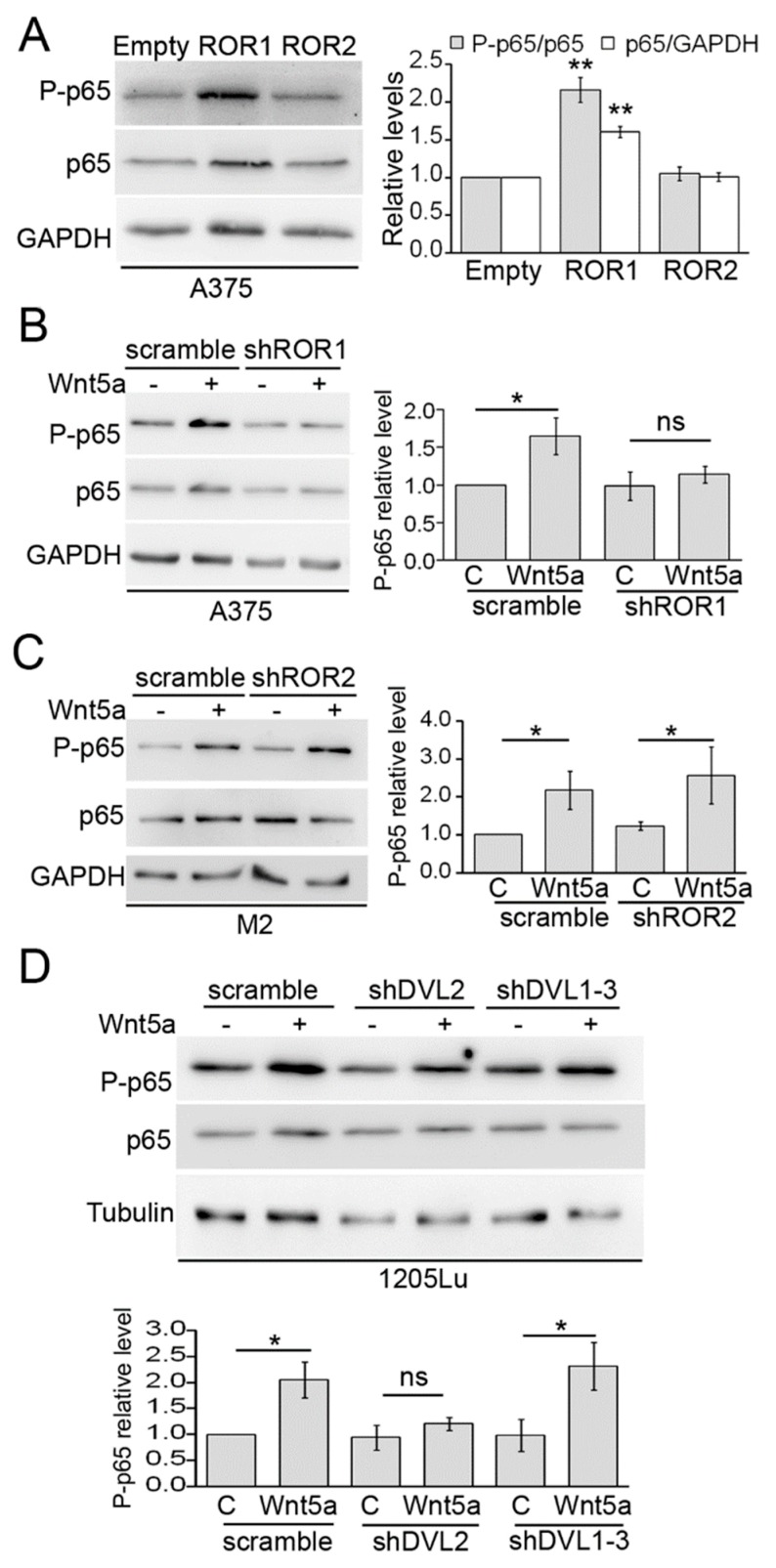
ROR1 and Dvl2 are required for p65 phosphorylation by Wnt5a. (**A**) Western blot analysis of A375 cells transduced with either ROR1 or ROR2. (**B**) A375 cells were transduced with a ROR1 shRNA or a scrambled shRNA and stimulated with either Wnt5a or control media for 30 min. The proteins extracts were blotted with the indicated antibodies. (**C**) M2 cells were transduced with a ROR2 shRNA or a scrambled shRNA and stimulated with either Wnt5a or control media for 30 min. The proteins extracts were blotted with the indicated antibodies. (**D**) 1205Lu cells were transduced with lentivirus encoding, either Dvl2 shRNA or Dvl1-3 shRNA, or a scramble sequence as a control, and stimulated with either Wnt5a or control media for 30 min. The proteins extracts were blotted with the indicated antibodies. GAPDH or Tubulin was used as the loading controls. The blots displayed are representative of three independent experiments. The bar graphs show the mean ± SD (from three independent experiments) of P-p65 levels normalized to the level of total p65 detected after stripping the membrane. Results are expressed as the fold change relative to control-treated cells (lane 1 in all panels). The statistical significance was tested by a student’s t-Test (treated sample vs. paired control in **B** –**D**) or ANOVA (**A**), followed by Dunnett’s Multiple Comparison Test, using log transformed FC values. * *p* < 0.05, ** *p* < 0.01, n = 3.

We next wanted to identify components of the Wnt non-canonical pathway that are required for Wnt5a-dependent p65 activation. First, we evaluated the involvement of the Wnt5a tyrosine kinase receptors ROR1 and ROR2, using both gain and loss of function approaches. The cell line A375 was stably transduced with lentivirus expressing either ROR1 or ROR2, and the overexpression of both genes was confirmed by western blot (Figure S3). Overexpression of ROR1 significantly increased both p65 and P-p65 levels (Figure 2A). In contrast, no effect was seen upon ROR2 overexpression (Figure 2A). To further substantiate these results, we stably silenced ROR1 and ROR2 expression by shRNA. Silencing of ROR2 was performed in the M2, since it expresses higher levels of ROR2, allowing a more accurate assessment of the efficiency of gene silencing (Figure S4) [44]. In line with our findings above, silencing of ROR1 abolished Wnt5a-induced p65 phosphorylation (Figure 2B). In contrast, ROR2 silencing did not affect the phosphorylation of p65 by Wnt5a (Figure 2C). These results are in agreement with previous observations showing that ROR1 overexpression mimics many of the effects elicited by Wnt5a [34]. We next assessed the participation of Dishevelled (Dvl), a key component of the Wnt pathway that relays Wnt signals from receptors to downstream effectors. After knocking-down Dvl isoforms by lentiviral shRNAs (Figure S5), we found that silencing of Dvl2, but not of Dvl1 and Dvl3, inhibited the phosphorylation of p65 induced by Wnt5a (Figure 2D). 

**Figure 3:**
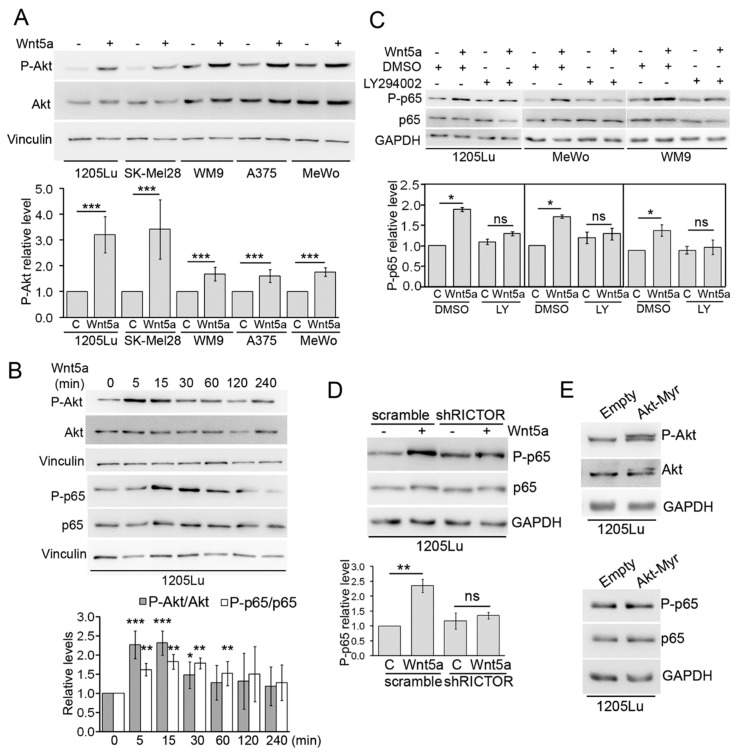
Activation of Akt is required for p65 phosphorylation by Wnt5a. (**A**) The indicated melanoma cell lines were stimulated with Wnt5a-CM (Wnt5a) and the proteins extracts were then blotted with the indicated antibodies. (**B**) 1205Lu cells were stimulated with Wnt5a-CM (Wnt5a) for the indicated times and the proteins extracts were then blotted with the indicated antibodies. (**C**) 1205Lu, MeWo, and WM9 cells were stimulated with Wnt5a-CM (Wnt5a) for 30 min in the presence of LY-294002 (10 µM). The proteins extracts were blotted with the indicated antibodies. (**D**) Cells transduced with a Rictor shRNA were stimulated with Wnt5a-CM (Wnt5a), and the proteins extracts were then blotted with the indicated antibodies. (**E**) Western blot analysis of 1205Lu cells transduced with Akt-Myr or an empty vector. The proteins extracts were blotted with the indicated antibodies. GAPDH or Vinculin were used as the loading controls. The blots displayed are representative of three independent experiments. The bar graphs show the mean ± SD (from three independent experiments) of P-p65 (**B**–**D**) and P-Akt (**A**,**B**) levels, normalized to the corresponding total protein and expressed as the fold change relative to control-treated cells (lane 1 in **B**,**D**). In panel A and C, each cell line has its own control-treated lane. Statistical significance in panels A,C,D,E,F was tested by a student’s t-Test (treated sample vs. paired control) (panels **A**,**C**,**D**) or ANOVA (panel **B**), followed by Dunnett’s Multiple Comparison Test, using log transformed FC values. * *p* < 0.05, ** *p* < 0.01, *** *p* < 0.001, n = 3.

We also evaluated the role of Akt, a protein kinase that was shown to be a target of Wnt5a in lung cancer [45] and melanoma cells [34]. In the five melanoma cell lines analyzed, stimulation with Wnt5a for 30 min significantly phosphorylates Akt at S473, a well-known marker of Akt activity (Figure 3A). The activation of Akt reached a peak between 5 and 15 min upon Wnt5a treatment, preceding the phosphorylation of p65 (Figure 3B) and leading to the speculation that Akt activity might be an upstream mediator of p65 phosphorylation by Wnt5a. To address this possibility, we used LY294002, a commonly used PI3K inhibitor that inhibits Akt phosphorylation by Wnt5a (Figure S6). Preincubation with LY294002 prevented p65 phosphorylation by Wnt5a in 1205Lu, MeWo, and WM9 cell lines (Figure 3C). These results suggest that the PI3K/Akt pathway is implicated in the activation of NF-κB by Wnt5a. To confirm this finding, we generated 1205Lu, stably expressing an shRNA against Rictor, a critical component of the mTORC2 complex responsible for Akt activation via S473 phosphorylation. As expected, Rictor shRNA inhibited Akt phosphorylation in 1205Lu cells (Figure S7) and prevented the phosphorylation of p65 induced by Wnt5a (Figure 3D). Interestingly, expression of a constitutively active form of Akt (Akt-Myr) did not increase P-p65 levels compared to control cells (Figure 3E), suggesting that, although Akt activity is required, it does not suffice to phosphorylate p65. The results above indicate that activation of NF-κB by Wnt5a requires upstream components of the Wnt5a pathway, such as ROR1, Dvl2, and Akt. 

### 3.3. Wnt5a Activates Canonical Components of the NF-κB Pathway

**Figure 4:**
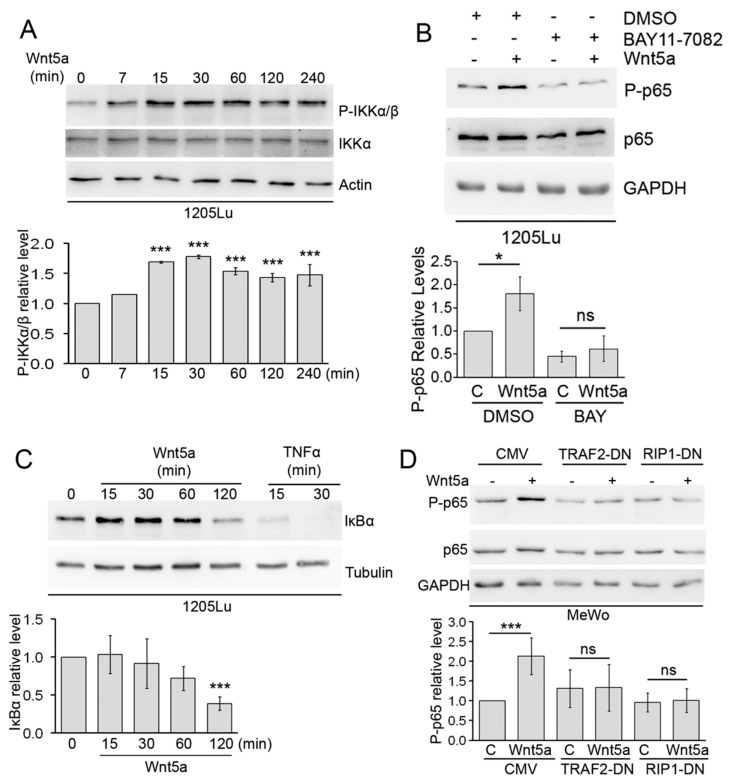
Wnt5a activates canonical components of NF-κB signaling. (**A**) 1205Lu cells were stimulated with Wnt5a-CM (Wnt5a) for the indicated times and the proteins extracts were then blotted with the indicated antibodies. (**B**) 1205Lu cells were stimulated with Wnt5a-CM (Wnt5a) for 30 min in the presence of BAY-117082 (10 µM). The proteins extracts were blotted with the indicated antibodies. (**C**) 1205Lu cells were stimulated with either Wnt5a-CM (Wnt5a) or TNFα (10 μg/mL) for the indicated times in the presence of cycloheximide (10 μg/mL). The proteins extracts were blotted with the indicated antibodies. (**D**) MeWo cells stably transfected with either TRAF2-DN, RIP-DN, or empty plasmid (CMV) were stimulated with either Wnt5a-CM (Wnt5a) or control media for 30 min. GAPDH, Tubulin, or Actin were used as loading controls. The blots displayed are representative of three independent experiments. The bar graphs show the mean ± SD (from three independent experiments) of P-IKKα/β (**A**), P-p65 (**B**,**D**), and IκBα (**C**) levels, normalized to total IKK (**A**), Tubulin (**C**), and total p65 (**B**,**D**), expressed as the fold change relative to control-treated cells (lane 1 in all panels). In panel **D**, each cell line has its own control-treated lane. The statistical significance in panels was tested by a student’s t-Test (treated sample vs. paired control, **B**,**D**) or ANOVA (**A**,**C**), followed by Dunnett’s Multiple Comparison Test, using log transformed FC values. *** *p* < 0.001, n = 3.

We next set to study the participation of known components of the upstream NF-κB cascade in the phosphorylation of p65 by Wnt5a. We first examined changes in IKK and IκBα, as they play critical roles in p65 regulation. During canonical NF-κB activation, IKK activation leads to IκBα phosphorylation and subsequent degradation [31]. Similarly, a significant increase in IKKα/β phosphorylation could be seen from 15 min following Wnt5a treatment (Figure 4A). The simultaneousness of p65 and IKK phosphorylation upon Wnt5a stimulation, and the fact that IKK has been regarded as the main kinase implicated in S536 phosphorylation of p65 upon TNFα, suggests the participation of IKK on Wnt5a-induced p65 phosphorylation. To confirm this possibility, we used the IKK inhibitor BAY-117082. As expected, BAY-117082 prevented the increase in p65 phosphorylation induced by Wnt5a (Figure 4B). In contrast, inhibition of GSK-3β (another kinase shown to phosphorylate p65) using LiCl [46] did not affect Wnt5a-induced p65 phosphorylation (Figure S8). In agreement with the increase in IKK phosphorylation, IκBα levels decreased after Wnt5a stimulation (Figure 4C). Although IκBα is not completely degraded, as occurred in the TNFα treatment, its levels were reduced significantly by more than 60%, 2 h after Wnt5a treatment (Figure 4C). To understand the involvement of more upstream components of the NF-κB pathway, we examined the role of TRAF2 and RIP. TRAF2 is an adaptor protein that plays critical roles in the signaling of the TNFα receptor superfamily members, whereas RIP is implicated in IKK complex activation on the canonical NF-κB pathway [31]. For this experiment, we used a dominant negative form of TRAF2 (TRAF2-DN) (Figure S9), lacking the amino-terminal RING finger domain (Δ1–87), and a dominant negative form of RIP (RIP-DN, aminoacids 559–671) that has been shown to block IKK activation during the canonical NF-κB pathway [47] (Figure S9). For this experiment, we used MeWo cells, since they presented the highest constitutive NF-κB activity among all our cell lines [48]. Expression of either TRAF2-DN or RIP-DN in MeWo cells abolished the induction of p65 phosphorylation by Wnt5a (Figure 4D). These results demonstrate that activation of the NF-κB pathway by Wnt5a shares many features with the canonical NF-κB pathway, including the participation of TRAF2 and RIP, IKK phosphorylation, IκBα degradation, and p65 phosphorylation.

### 3.4. Wnt5a-Induced NF-κB Transcriptional Activity

**Figure 5:**
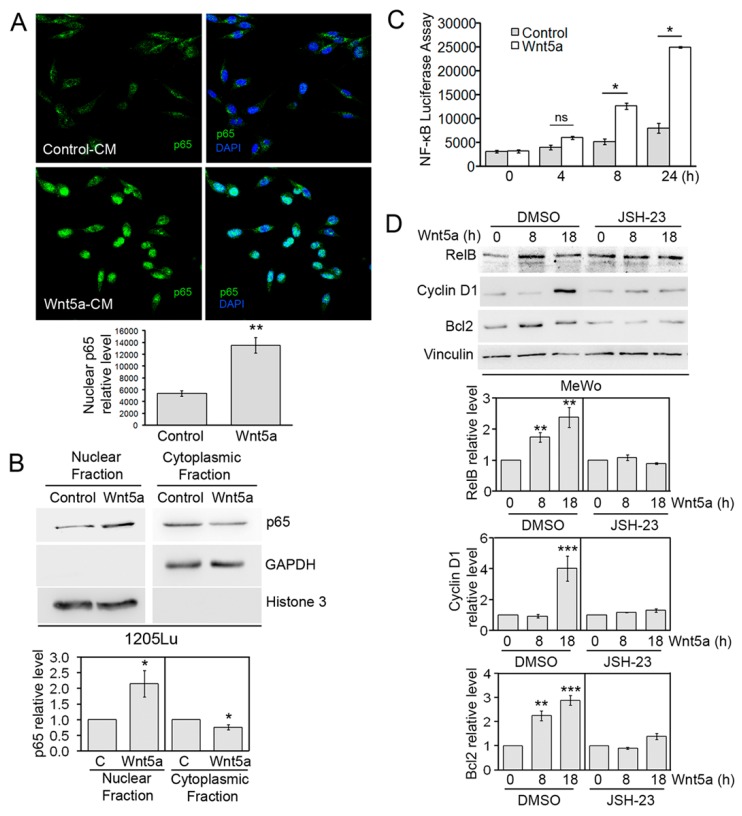
Wnt5a induces p65 translocation and NF-kB transcriptional activity. (**A**) SK-Mel2 cells were stimulated with Wnt5a-CM or Control-CM, and the localization of p65 was determined by immunofluorescence (Left panels. FITC, green). Panels on the right show FITC images merged to DAPI staining of DNA (blue). Areas of overlap between p65 and the DNA appear in light blue. The bar graphs show the mean ± SD (from three independent experiments) of the nuclear fluorescent p65 staining intensity of 10 fields per treatment and experiment. The statistical significance was tested by a one-tailed student t-Test, **p < 0.01, ns: not significant, n = 3. (**B**) Nuclear and cytoplasmic fractions obtained from 1205Lu cells stimulated with Wnt5a-CM (Wnt5a) were blotted with p65 antibodies. Histone 3 and GAPDH were used as nuclear and cytosolic markers. The blots displayed are representative of three independent experiments. Bar graphs show the mean ± SD (from three independent experiments) of p65 levels, normalized to either Histone 3 or GAPDH and expressed as the fold change relative to control-treated cells. (**C**) M2 cells were transfected with a NF-κB synthetic luciferase reporter construct (pNF-κB-Luc) and stimulated with Wnt5a-CM (Wnt5a) or control media for different time intervals. Results are shown as the mean ± SD. The statistical significance was tested by a student’s t-Test. **p ˂ 0.01. (**D**) MeWo cells were stimulated with Wnt5a-CM (Wnt5a) or control media for different time intervals in the presence of JSH-23 (10 µM) or DMSO. The protein extracts were blotted with the indicated antibodies. Vinculin was used as the loading control. The blots displayed are representative of three independent experiments. The bar graphs show the mean ± SD (from three independent experiments) of the levels of RelB, Cyclin D1, and Bcl2, normalized to Vinculin levels and expressed as the fold change relative to control-treated cells (0 h time-point). The statistical significance was tested by ANOVA, followed by Dunnett’s Multiple Comparison Test, using log transformed FC values. * *p* < 0.05, ** *p* < 0.01, *** *p* < 0.001, n = 3.

Activation of the NF-κB pathway involves translocation of p65 to the nucleus, where binding to DNA and transcriptional regulation occurs. Therefore, we wanted to assess changes in the cellular localization of p65 proteins upon Wnt5a treatment by using fluorescent microscopy. Following fixation and permeabilization, 1205Lu and SK-Mel2 cells were stained with an antibody against p65 and counterstained with DAPI. After Wnt5a treatment, the cells displayed a mostly nuclear staining pattern that markedly differed from the diffuse staining observed throughout the cell, following stimulation with control CM (Figure 5A and Figure S10). Quantification of these images revealed a significant increase in nuclear p65 upon Wnt5a treatment (Figure 5A). We next evaluated changes in p65 localization by subcellular fractionation and western blotting. Treatment with Wnt5a resulted in the accumulation of p65 in the nuclear fraction with a concomitant reduction in the cytoplasmic fraction (Figure 5B). The presence and purity of the subcellular fractionation were assessed by using the cytosolic marker GAPDH and the nuclear marker Histone 3. Since Wnt5a induced p65 nuclear translocation and phosphorylation of S536, and the latter was shown to enhance NF-κB transactivation through increased p65 acetylation and CBP/p300 binding [31], we wanted to determine whether Wnt5a activates NF-κB transcriptional activity. To this end, a luciferase reporter assay was performed. M2 melanoma cells were transfected with an NF-κB synthetic luciferase reporter construct and stimulated with Wnt5a for different time intervals. NF-κB luciferase activity significantly increased in a time-dependent manner, reaching a 3-fold increase at 24 h following treatment (Figure 5C). To confirm this finding, we evaluated changes in expression levels of three known NF-κB target genes, following Wnt5a stimulation. In agreement with previous results, Wnt5a significantly increased the expression levels of RelB, Cyclin D1, and Bcl2 (Figure 5D). Since the expression of these genes can also be regulated by other pathways, we determined the specific contribution of NF-κB to the transcription of these genes by using JSH-23, a selective inhibitor of p65 nuclear translocation and its transcriptional activity [49]. Our data show that, in the presence of JSH-23, Wnt5a no longer augmented RelB, Cyclin D1, and Bcl2 levels (Figure 5D). Upregulation of Cyclin D1 and Bcl2 by Wnt5a was also observed in melanocytes (Figure S11). Altogether, these results indicate that Wnt5a stimulates NF-κB transcriptional activity.

### 3.5. Wnt5a Induced Secretion of Cytokines and Chemokines by Melanoma Cells

**Figure 6:**
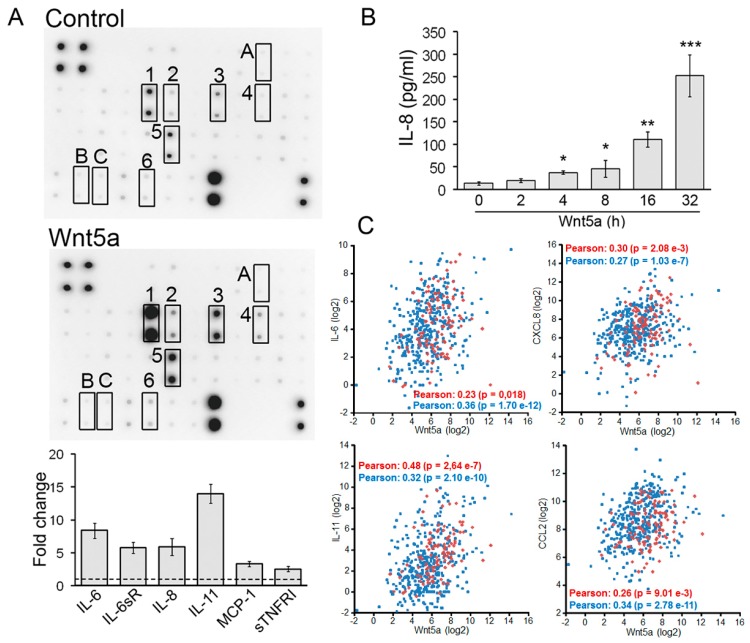
Wnt5a induced secretion of pro-inflammatory cytokines by melanoma cells. (**A**) Representative image showing the human inflammatory protein array probed with proteins from MeWo cells stimulated with Wnt5a. Culture supernatants from MeWo cells treated with Wnt5a-CM (Wnt5a) or Control-CM (Control) for 32 h were assayed using a Human cytokine array. Each cytokine is represented by duplicate spots. Cytokines that were induced by Wnt5a are indicated: 1, IL-6; 2, IL-6sR; 3, IL-8; 4, IL-11; 5, MCP1; 6, sTNFRI; A, INFγ; B, TGFβ; C, and TNFα. Positive and negative controls are in the upper left (n = 4) and lower right (n = 2) corners, respectively. The average net signal intensity of each pair of cytokine spots was determined using ImageJ and expressed as the fold change relative to control-treated cells. The dotted line denotes a fold-change of 1. (**B**) IL-8 levels were determined by ELISA in culture supernatants from MeWo cells stimulated with Wnt5a-CM (Wnt5a) for the indicated times. Results are shown as the mean ± SD * *p* < 0.05, ** *p* < 0.01, *** *p* < 0.001, n = 3, ANOVA, followed by Dunnett’s Multiple Comparison Test. (**C**) Wnt5a expression levels correlate with the expression levels of IL-6, IL-8, IL-11 and MCP-1 in melanoma. Co-expression of Wnt5a with IL-6, IL-8 (CXCL8), IL-11, and MCP-1 (CCL2) in primary (red symbols) and metastatic (blue symbols) samples from The Cancer Genome Atlas (TCGA) public datasets for melanoma (skin cutaneous melanoma (SKCM), 479 patients). TCGA RNA-seq data were extracted using the cBioportal database. The value of Pearson’s correlation coefficient, as well as the *p* value, were generated by the cBioportal database to measure the correlation of gene co-expression.

Since the NF-κB pathway is a master regulator of inflammation, we wanted to assess whether Wnt5a can increase the expression of cytokines and chemokines by melanoma cells. To this end, culture supernatants from MeWo cells stimulated with Wnt5a for 32 h were evaluated using a human cytokine array. This analysis revealed that Wnt5a enhanced the expression of several cytokines and chemokines, including IL-6, IL-6 soluble receptor (IL-6sR), IL-8 (CXCL8), IL-11, TNFα soluble receptor I (sTNFRI), and MCP-1 (CCL2), with a fold increase over control-treated cells between 2 and 14 (Figure 6A). Importantly, Wnt5a did not upregulate TGFβ1, INFγ, or TNFα, ruling out the possibility that the rise on the levels of the above-mentioned cytokines and chemokines was mediated by those classical factors. To validate the effect of Wnt5a on cytokine/chemokine production, we determined changes in IL-8 levels by ELISA. IL-8 levels significantly rose at 4 h upon Wnt5a treatment and further increased over time, reaching a 19-fold increase at 32 h after Wnt5a treatment (Figure 6B). Of note, this increase in the IL-8 level was much larger than the seven-fold increase detected by the array, suggesting that this assay might be underestimating the upregulation of cytokines and chemokines.

The increase in IL-8 release following Wnt5a treatment was also observed in 1205Lu and SK-Mel28 cell lines (Figure S12). To evaluate the relationship between Wnt5a and these cytokines/chemokines in melanoma samples, we used cBioPortal [50] to determine the co-expression of some of these genes with Wnt5a in TCGA public datasets for melanoma (SKCM, 479 patients). In agreement with our results, the expression of IL-6, IL-8 (CXCL8), IL-11, and MCP-1 (chemokine ligand 2 (CCL2) significantly correlated with that of Wnt5a in metastatic melanoma tumors (Figure 6C, blue symbols). With the exception of IL-6, the level of these cytokines in primary melanoma tumors also significantly correlated with that of Wnt5a (Figure 6C, red symbols). These results demonstrate that activation of NF-κB by Wnt5a stimulates the production of cytokines and chemokines by melanoma cells.

### 3.6. Melanoma Cells Present a Wnt5a Autocrine Circuit to Activate NF-κB 

**Figure 7:**
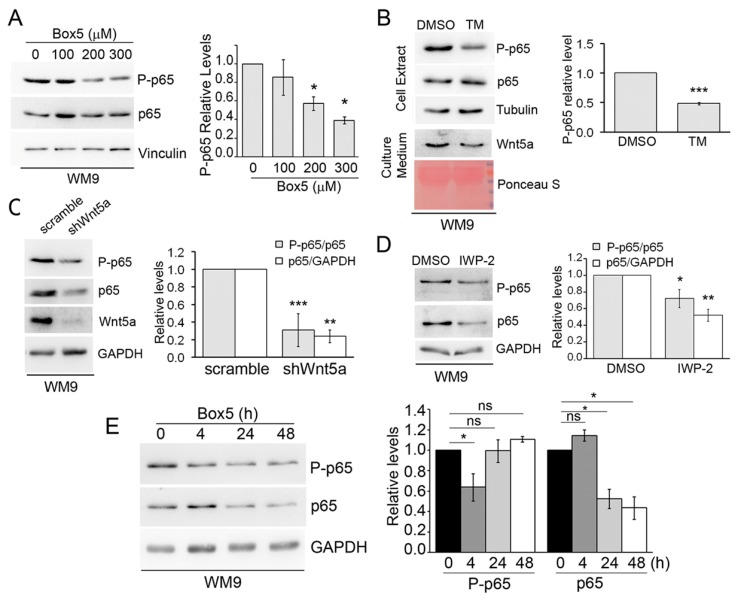
Melanoma cells stimulate autocrine activation of p65. (**A**) Western blot analysis of WM9 cells incubated with the indicated concentrations of Box5 or DMSO for 4 h. (**B**) Western blot analysis of WM9 cells incubated with Tunicamycin (4 µM) or DMSO for 12 h. Both cell extracts and conditioned media were blotted with the indicated antibodies. (**C**) Western blot analysis of WM9 cells incubated with IPW-2 (20 µM) or DMSO for 72 h. The proteins extracts were blotted with the indicated antibodies. (**D**) WM9 cells were stably transduced with lentivirus, encoding Wnt5a shRNA or a scramble sequence as a control. The protein extracts were blotted with the indicated antibodies. (**E**) Western blot analysis of WM9 cells incubated with Box5 (200 µM) for the indicated times. Vinculin, Tubulin, or GAPDH were used as loading controls. The blots displayed are representative of three independent experiments. The bar graphs show the mean ± SD (from three independent experiments) of P-p65 levels, normalized to the level of total p65, detected after stripping the membrane. In panels **C**, **D**,**E**, to evaluate changes in total p65, the data were normalized to GAPDH levels. Results are expressed as the fold change relative to control-treated cells (lane 1). The statistical significance was tested by a atudent’s t-Test (**B**–**D**) or ANOVA, followed by Dunnett’s Multiple Comparison Test (**A**,**E**), using log transformed FC values. * *p* < 0.05, ** *p* < 0.01, *** *p* < 0.001, n = 3.

While all the melanoma cell lines assayed showed activation of p65 phosphorylation by Wnt5a, some of them, such as 1205Lu, WM983A, and WM9, displayed a moderate effect with a fold increase between 2 and 3. Coincidentally, these cell lines present high levels of constitutive p65 phosphorylation (Figure 1A,D) and were previously found to abundantly express Wnt5a [34]. These observations raised the possibility that the high basal levels of phosphorylated p65 in these cells might be due to a Wnt5a autocrine loop. This could explain why the stimulation with Wnt5a-CM just induced a modest increase of the p65 phosphorylation in these cells. To test this possibility, we measured changes in phosphorylated p65 levels upon inhibition of endogenous Wnt5a in WM9 cells by both pharmacological and genetic approaches. Incubation of WM9 cells for 4 h with Box5, a Wnt5a-derived hexapeptide that antagonizes Wnt5a [51], dose-dependently inhibited p65 phosphorylation (Figure 7A). Similarly, Tunicamycin, a bacterial compound that decreases cellular expression and secretion of Wnt5a by inhibiting its N-linked glycosylation [52], significantly diminished basal p65 phosphorylation of WM9 cells after 12 h (Figure 7B). These results demonstrate that inhibition of endogenous Wnt5a-decreased basal phosphorylation of the critical S536 residue in p65 in melanoma cells. To confirm these results, we inhibited Wnt5a expression by stable transduction of WM9 cells with a lentivirus encoding a Wnt5a shRNA. This experiment revealed that Wnt5a knock-down significantly reduced both phosphorylated and total p65 levels (Figure 7C). The unexpected decrease in total p65 levels was not an artifact of generating a stable cell line, since protein extracts obtained from WM9 cells 48 h after transduction with the Wnt5a shRNA yielded the same result (Figure S13). The downregulation of both basal phosphorylated and total p65 levels were also observed upon inhibiting endogenous Wnt5a by an incubation of 72 h with IWP-2 (Figure 7D). These results show that inhibition of endogenous Wnt5a decreased basal p65 phosphorylation, but as the inhibition prolongs in time (i.e., more than 24 h), total expression of the p65 protein is also compromised. To attempt to observe these two effects in the same experimental setting, we treated WM9 cells with Box5 for different time-points. As previously shown (Figure 7A), the early effect of Wnt5a inhibition just reduced the levels of phosphorylated p65, whereas at later time points it also reduced total p65 levels (Figure 7E). Altogether, these results reveal that Wnt5a, when expressed by the cells, triggers an autocrine loop that activates the NF-κB pathway. Along this line, interruption of this loop by inhibition of Wnt5a can drastically inhibit the NF-κB pathway.

## 4. Discussion

Wnt signaling is known to have roles both in melanoma initiation and progression. Wnt5a has garnered interest in melanoma after the observation that it is highly expressed in metastatic melanoma and that its high expression correlates with disease progression and decreased progression-free and overall survival [18]. Later studies described the role of Wnt5a in regulating cell motility and invasion [53,54]; a concept that was confirmed after its identification as one of the 45 genes defining an invasive signature in melanoma cells [55]. Since then, the investigation of its role in invasion and metastasis has been a staple of Wnt5a research in melanoma and other tumor types. 

**Figure 8:**
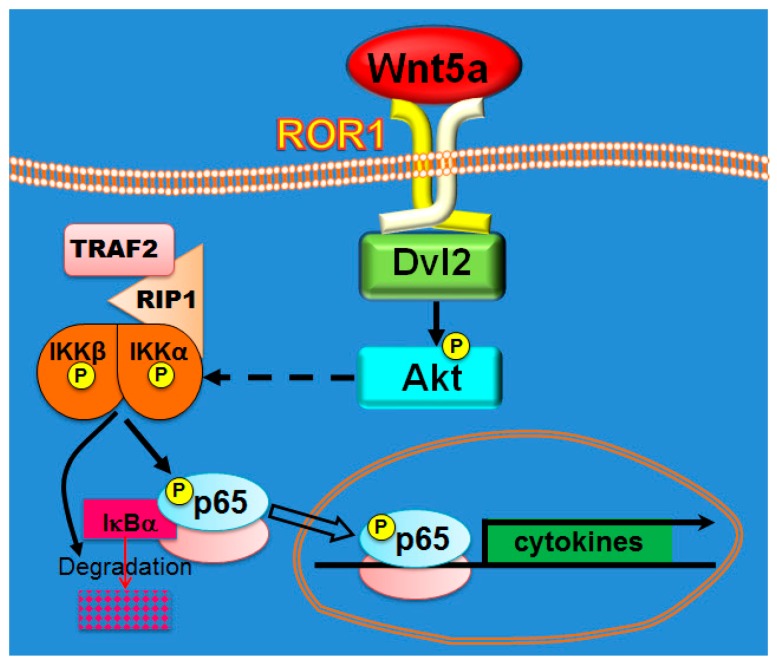
Model of p65 activation by Wnt5a. Wnt5a stimulates Akt phosphorylation with the participation of ROR1 and Dvl2. Akt induces activation of the IKK complex, which in turn phosphorylates S536 of p65 and induces IκBα degradation. TRAF2 and RIP1 participate in this process. Upon IκBα degradation, p65 translocates to the nucleus and induces transcription of cytokines and other NF-κB target genes.

The participation of Wnt5a in several inflammatory diseases is well known, but its role in tumor-promoting inflammation has been scarcely and fragmentarily studied. It has been shown that Wnt5a can promote cytokine expression in cancer cells [27,28,56,57,58]. However, none of these studies explored the mechanisms underlying cytokine regulation by Wnt5a. Given the known role of NF-κB on regulating cytokine expression, it is easy to envision the involvement of the NF-κB pathway in this process following Wnt5a stimulus. Activation of NF-κB by Wnt5a was described in several cell types, usually immune cells [59]. But again, the existence of a Wnt5a-NF-κB connection has not been conclusively demonstrated in cancer cells. The only references in such regard is that activation of NF-κB by Wnt5a was shown to be required for BMP-6 mRNA and MMP7 upregulation in LNCaP [60] and breast cancer cells [61], respectively. In line with the notion above, regulation of inflammation has not been considered as a frequent and important effect of Wnt5a in cancer [5,6,7,8,9,62,63]. Our work shows NF-κB activation by Wnt5a, identifies critical intermediaries of this process, and demonstrates that Wnt5a stimulates secretion of cytokines/chemokines in melanoma cells. For this crosstalk to occur, the participation of components of both the Wnt5a and the NF-κB pathways is required (Figure 8). In relation with the former pathway, we described the important role of both the tyrosine kinase receptor ROR1 and the Wnt signalosome core protein Dvl2. The possibility of a ROR1-NF-κB axis was raised by Fukuda et al. and Díaz –Horta et al., who described that ROR1 overexpression increased NF-κB reporter activity in HEK293T cells [64,65]. These findings and data from our laboratory ([34] and this manuscript) suggest that ROR1 overexpression mimics many of the effects elicited by Wnt5a. Similarly, Dvl3 has been implicated in p65 nuclear translocation of p65 via a p38-dependent activation mechanism in lung cancer cells [66].

Downstream of its plasma membrane receptors and the scaffold proteins Dvls, Wnt5a signaling can activate several pathways [5,6,7,8,9]. In recent years, we and others demonstrated that Akt is a novel and important target of Wnt5a [34,45,67]. Here, we show that activation of Akt is required for the NF-κB pathway activation by Wnt5a. This finding is in agreement both with the correlation found between active Akt and active p65 in biopsies from melanoma patients [68] and with the observed deactivation of NF-κB upon Akt inhibition seen in several tumor types, including melanoma [68,69,70]. The link between Akt and NF-κB has been extensively documented and many publications even refer to a “PI3K/Akt/NF-κB pathway”, although many features of this “pathway” have been poorly defined [70,71]. Although there is a consensus that the regulatory effect of Akt on p65 is mediated by S536 phosphorylation, the identity of the implicated kinase is still not clear. It has been often assumed that this process might be directly mediated by Akt. However, the amino acid sequence surrounding the S536 (GDEDFSSIADMDF) does not correspond to the consensus Akt site (RXRXXS/TF/L), an issue that has been frequently overlooked. In line with this observation, other groups have proposed other protein kinases, such as mTOR, IKK, or p38, as implicated in this reaction [72,73]. Our results support the role of IKK on Wnt5a-dependent p65 phosphorylation. This, together with the participation of TRAF2 and RIP, suggests that NF-κB activation by Wnt5a shares many features with the canonical NF-κB activation. Concerning the events leading to the phosphorylation of IKK, they are still a matter of debate in the NF-κB field and have not been explored in the present manuscript. To explain the phosphorylation of IKK upon TNFα, both IKK kinases (i.e., TAK1, RIP1, MEKK3) and IKK trans-autophosphorylation have been described [31,74]. Downstream of IKK phosphorylation, activation of NF-κB by both TNFα and Wnt5a leads to phosphorylation-dependent degradation of IκBα, although the process triggered by Wnt5a is much slower and less efficient than that triggered by TNFα. Our data show that IκBα is just partly degraded 2 h upon Wnt5a treatment, a situation similar to that observed upon other stimuli activating NF-κB, such as CD40 [75]. This difference is not trivial and might account for differences in gene expression programs induced by different stimuli that activate the same IKK-IκB-NF-κB signaling module [32]. 

The elevated Wnt5a expression seen in both melanoma metastasis and melanoma cell lines with an invasive phenotype made it feasible to study the Wnt5a/NF-κB crosstalk by using strategies based on the inhibition of endogenous Wnt5a. This approach has led to confirm Wnt5a´s ability to phosphorylate p65, but also allowed us to determine that sustained inhibition of Wnt5a signaling affects p65 expression as well. In contrast to the detailed knowledge we have about post-translational modifications regulating p65/RelA activity and localization, little is known about the regulation of its transcription. These observations position the Wnt5a pathway as a major regulator of NF-κB, at least in melanoma cells. Future studies will allow characterizing, which of the many pathways activated by Wnt5a controls p65 expression.

Wnt5a has been shown to stimulate cytokine/chemokine secretion in different types of cells [76,77,78,79]. Many of these studies have focused on macrophages that use their Wnt5a to promote the release of the pro-inflammatory cytokines required to sustain macrophage’s inflammatory response [26]. In contrast, just a few studies have described the upregulation of cytokines by Wnt5a in tumor cells [27,28]. In melanoma, Wnt5a was associated with the release of IL-6, VEGF, and MMP2 [58]. In the present work, we have used an unbiased approach to explore the production of cytokines and chemokines in further depth by using a microarray for simultaneous detection of 40 human cytokines. Our results reveal that Wnt5a stimulates melanoma cells to secrete IL-6, IL-8, IL-11, MCP-1, IL-6sR, and sTNFRI. The identification of these proteins offers an insight into the immunomodulatory functions of Wnt5a. IL-6, IL-6sR, sTNFRI, and IL-11 are pro-inflammatory cytokines that contribute to angiogenesis, cell proliferation, tumor cell survival, exhaustion of effector T cells, and therapeutic resistance [80,81]. Wnt5a also promotes the release of the chemokines IL-8 and MCP-1. Besides promoting tumor progression by acting on both tumor and non-tumor cells from the tumor microenvironment, these proteins strongly chemoattract monocytes (MCP-1) and neutrophils and T cells (IL-8) to both primary and metastatic tumors [82]. Monocyte-derived tumor-associated macrophages are particularly abundant in the tumor microenvironment and generally play a pro-tumoral role. During metastasis, macrophages promote tumor cell extravasation, survival, and immunosuppression [83]. Neutrophils also favor tumor growth through the formation of NETs Neutrophil extra-cellular traps (NETs), the release of reactive oxygen species (ROS), and the promotion of immunosuppression [84]. Our observation that links Wnt5a with cytokine/chemokine secretion and immunosuppression is in agreement with a recent finding that suggested a role of Wnt5a in resistance to anti-PD-1 therapy. Hugo and coworkers described that Wnt5a is part of a transcriptional signature found in PD-1 innately resistant tumors and referred to as IPRES or Innate anti-PD-1 RESistance [85]. Our findings provide a biological significance for the high levels of Wnt5a in patients with IPRES, which would contribute to the generation of a pro-inflammatory and immunosuppressive tumor microenvironment that precludes the effect of anti-PD-1 therapy. It remains to be determined whether Wnt5a is a major contributor to the phenomenon of innate resistance to PD-1 or just a biomarker of this process. The former possibility seems plausible since (i) the IPRES signature contains a large number of Wnt signaling pathway genes, (ii) it also contains immunosuppressive genes, such as VEGF, and chemokines, such as IL-8, CCL2, CCL7, and CCL8, which were shown by us and others to be regulated by Wnt5a ([56,57,58,76,77] and this study), and (iii) the effects of Wnt5a secreted by melanoma cells will likely persist and be amplified in the tumor microenvironment, thanks to autocrine positive feedback loops with NF-κB [86], STAT3 [86,87], IL-6 [88], and paracrine effects over other cells (i.e., macrophages). In summary, we show that Wnt5a, initially studied for its role in tumor migration and invasion, activates NF-κB and exerts an immunomodulatory role, further establishing Wnt5a as a promising therapeutic target for melanoma.

## Supplementary Materials

The following are available online at https://www.mdpi.com/2073-4409/8/9/1060/s1. Figure S1: IWP-2 prevents Wnt5a secretion, Figure S2: Wnt5a induced p65 phosphorylation in melanocytes, Figure S3: A375 cell lines stably expressing ROR1 and ROR2, Figure S4: Silencing of ROR1 and ROR2 in melanoma cell lines, Figure S5: Silencing of Dvl proteins in 1205Lu cells, Figure S6L Effect of LY294002 on Akt phosphorylation, Figure S7: Silencing of Rictor in 1205Lu cells, Figure S8: LiCl does not inhibit p65 phosphorylation by Wnt5a, Figure S9: Assessment of DN-TRAF2 and DN-RIP expression, Figure S10: Wnt5a induces p65 nuclear translocation, Figure S11: Wnt5a upregulates NF-kB targets on melanocytes, Figure S12: Wnt5a stimulates IL-8 secretion, Figure S13: Wnt5a silencing decreases p65 level.
